# Glycolysis and Heavy Menstrual Bleeding

**DOI:** 10.1007/s43032-022-01150-3

**Published:** 2022-12-27

**Authors:** Kalle T. Rytkönen

**Affiliations:** 1 Institute of Biomedicine, Research Centre for Integrative Physiology and Pharmacology, University of Turku, Kiinamyllynkatu 10, 20014 Turku, Finland; 2 Turku Bioscience Centre, University of Turku and Åbo Akademi University, Tykistökatu 6, 20520 Turku, Finland

**Keywords:** Uterus, Endometrium, Glycolysis, Heavy menstrual bleeding, Decidualization, Hypoxia

## Abstract

Menstrual cycle is a major determinant in female reproductive health. In a recent report, Mao et al. (2022) associated deficient glycolysis with heavy menstrual bleeding. This commentary summarizes these recent findings and the importance of glycolysis and decidualization in endometrial function. It will also discuss if in the light of the recent findings menstrual bleeding is better conceived as a primary endometrial disorder inherent to endometrium or as a secondary endometrial disorder caused by other endometrial conditions.

Female reproductive health is dependent on the cyclic menstruation-related renewal of the uterine endometrium. In each menstrual cycle, the endometrial cells differentiate (decidualize) to prepare the uterus either for pregnancy or for menstrual discharge. This hormonally fine-tuned cycle consists of proliferative, secretory, and menstrual phases. Deviations in these phases may cause reproductive disorders and conditions including abnormal menstrual bleeding or heavy menstrual bleeding (HMB) that affect up to one-third of reproductive-aged women [1].

The renewal and regrowth of the endometrium require considerable energetic input. Proliferative endometrium relies relatively more on aerobic energy production, whereas during the secretory phase, larger proportion of the energy is produced anaerobically via glycolysis [2]. Concordantly, glycolysis has been shown to be necessary for endometrial stromal decidualization [3] that enables implantation/pregnancy or menstruation. However, the importance of glycolysis for menstruation has remained understudied. Recently, a study by Chenyu Mao, Xishi Liu, and Sun-Wei Guo reports that decreased glycolysis is associated with heavy menstrual bleeding [4]. To prove this hypothesis, the authors utilized a series of mouse experiments, cell culture experiments, and studies of relevant human samples.

In order to study the correlation between glycolysis and menstruation, Mao et al. used mouse model to mimic human menstruation and repair [4]. Mice do not menstruate, but with hormone stimulation, human-like menses can be induced also in mice. More specifically, mice were first treated with estrogen to induce endometrial proliferation and progesterone to induce decidualization. Subsequently, a drop in progesterone levels in mice-induced endometrial breakdown and bleeding similarly as in human cycle. The authors observed that the protein levels of glycolytic genes, including enzymes and transporters, are increased in the decidualized uterus compared to non-decidualized uterus. By using inhibitor of glycolytic enzyme hexokinase 2 (HK2) and immunohistochemistry, they demonstrated that inhibition of glycolysis reduced inflammatory response and impaired the menstruation-associated repair process of the endometrium. This also resulted in increased blood loss measured using cotton balls inserted and collected after progesterone withdrawal. Additionally, in a human endometrial epithelial cell line, the same inhibitor treatment resulted in reduced cell proliferation and migration.

Earlier studies have shown that hypoxia occurs in the endometrium during menstruation. With the same mouse model, it was shown that menstruation-associated hypoxia stabilizes the levels of the hypoxia inducible factor 1alpha (HIF-1alpha) and that this transcription factor is essential for proper endometrial repair during menstruation [5]. Generally, HIF-1alpha is a well-studied regulator of hypoxia adaptation and evolutionary conserved inducer of glycolytic genes. Now, in an additional simulated mouse experiment, Mao et al. study the relationship between HIF-1alpha, glycolysis, and endometrial repair. By using inhibitors of HIF-1alpha and HK2 (glycolysis), the authors observed that inhibiting glycolysis effectively disrupted menstrual repair even in the presence of hypoxia dependent HIF-1alpha stabilization. This further enforced the conclusion of importance of specifically glycolytic metabolism, not just hypoxia per se, in the menstrual repair.

Lastly, the authors collected samples from patients who had experienced heavy menstrual bleeding and measured the amount of blood loss was with menstrual pictogram in absorbing menstrual towels. Patients that had more than 100 mL of menstrual blood loss were defined as excessive bleeding group, and those who had less than 100 mL of menstrual blood loss were defined as control group. The authors discovered reduced immunostaining of HK2 indicating reduced glycolysis in women with excessive menstrual bleeding suggesting that defective glycolysis causes heavy menstrual bleeding also in humans.

On a more fundamental level, it still remains unresolved whether endometrial disorders that lead to heavy menstrual bleeding are primary endometrial disorders or secondary endometrial disorders. In a primary endometrial disorder, defects in endometrial tissue or cells directly functionally cause the bleeding disorder, whereas in a secondary endometrial disorder physically measurable condition such as adenomyosis, uterine polyps and leiomyoma (uterine fibroids) cause heavy bleeding as a secondary effect [1].

In Mao et al. study, the patient samples were adjacent to adenomyosis lesions. Adenomyosis is a condition where endometrial cells penetrate to the myometrium, the muscle layer outside the uterus, and generate lesions. The proximity of the samples to adenomyosis lesions suggests that the described defect in glycolysis could be specific to adenomyosis-induced heavy menstrual bleeding. On the other hand, considering the experiments conducted in mouse, in vitro and in human samples as a whole indicates that glycolysis per se is important for normal menstrual repair, which in turn suggests that glycolysis defect would be a primary disorder.

Downregulation of endometrial glycolytic genes such as HK2 has also been observed in preeclampsia [6]. Could this observation advice whether defected glycolysis causing heavy bleeding should be viewed as primary or secondary disorder? In preeclampsia, that is a pregnancy disorder characterized by high maternal blood pressure, downregulation the endometrial HK2 was associated with defective decidualization and considered as a potential contributor to preeclampsia [6]. Similarly, in the study by Mao et al., the observed downregulation of inflammation and reduced cell migration is also a general sign of decidualization defect. Furthermore, non-pregnant menstruation and parturition that ends pregnancy have several molecular similarities [7]; both are preceded by progesterone-induced decidualization and subsequent progesterone withdrawal. Thus, in both cases, the downregulation of glycolytic enzymes can be conceived as evidence of decidualization defect, which would also include the view of endometrial glycolysis defect as primary endometrial disorder originating from the endometrial cell functions, and not as a secondary effect from physically measured conditions such as adenomyosis. On the other hand, adenomyosis have also been associated with higher occurrence of preeclampsia [8]—making it, again, more difficult to evaluate the causality. In any case, collectively, the evidence points out that a well-balanced glycolytic drive is essential for healthy endometrial function.

Currently, hormonal interventions are the most common treatments for heavy menstruation, including levonorgestrel-releasing intra-uterine devices. The usefulness of these has become evident during last decades, but for some patients, other than hormonal medications may be beneficial. The results from Mao et al. suggest that non-hormonal treatments, potentially based on enforcing the HIF pathway or glycolysis to alleviate heavy menstruation, are plausible.

## Data Availability

Discussed data available in the original publications.
